# Statistical Methods for Linking Health, Exposure, and Hazards

**DOI:** 10.1289/ehp.7145

**Published:** 2004-08-03

**Authors:** Frances Jean Mather, LuAnn Ellis White, Elizabeth Cullen Langlois, Charles Franklin Shorter, Christopher Martin Swalm, Jeffrey George Shaffer, William Ralph Hartley

**Affiliations:** ^1^Department of Biostatistics, Academic Information Systems,; ^2^Department of Environmental Health Sciences, Center for Applied Environmental Public Health, and; ^3^Academic Information Systems, Tulane University School of Public Health and Tropical Medicine, New Orleans, Louisiana, USA

**Keywords:** Bayesian modeling, data linkage, exposure, GIS, hazards, health outcome data, statistical methods

## Abstract

The Environmental Public Health Tracking Network (EPHTN) proposes to link environmental hazards and exposures to health outcomes. Statistical methods used in case–control and cohort studies to link health outcomes to individual exposure estimates are well developed. However, reliable exposure estimates for many contaminants are not available at the individual level. In these cases, exposure/hazard data are often aggregated over a geographic area, and ecologic models are used to relate health outcome and exposure/hazard. Ecologic models are not without limitations in interpretation. EPHTN data are characteristic of much information currently being collected—they are multivariate, with many predictors and response variables, often aggregated over geographic regions (small and large) and correlated in space and/or time. The methods to model trends in space and time, handle correlation structures in the data, estimate effects, test hypotheses, and predict future outcomes are relatively new and without extensive application in environmental public health. In this article we outline a tiered approach to data analysis for EPHTN and review the use of standard methods for relating exposure/hazards, disease mapping and clustering techniques, Bayesian approaches, Markov chain Monte Carlo methods for estimation of posterior parameters, and geostatistical methods. The advantages and limitations of these methods are discussed.

The environment plays an important role in health and human development. Acute effects from exposure to environmental contaminants, such as pesticide poisoning, are well recognized, but the environmental link to most chronic diseases remains unclear. Researchers have linked exposure to specific environmental hazards with a health effect, such as benzene and leukemia. Other associations are suspect, such as exposure to mixtures of drinking water disinfection by-products and bladder cancer. In other cases, linkages between environmental agents, individually or as mixtures, and health outcomes lack epidemiologic evidence and are postulated from laboratory animal studies. The Pew Environmental Health Commission (2000) calls the lack of information linking environmental hazards and chronic disease the “environmental health gap.” To address this gap, the Centers for Disease Control and Prevention (CDC) established the National Environmental Public Health Tracking Network (EPHTN; CDC 2003a), which is developing the infrastructure, resources, and methods for assembling and using available environmental hazard, exposure, and health outcome data (HOD). This initiative presents great methodologic challenges such as using existing data in new ways and for purposes other than for which they were collected; expanding the limited guidance for using available statistical methods to analyze and link data; and closing gaps in methodology for linking disparate data sets. Despite the challenges, great opportunities exist to forge partnerships to make data more available, develop standards to facilitate data exchange, and analyze data to describe the impact of environmental hazards on human health. However, without defining the appropriate rules for data linkage, indiscriminate linking may lead to erroneous conclusions. This highlights the need to understand each data set, articulate the uses and limits of each data set, and standardize methods for using the data.

## Fundamental Premise for Linking Data

Fundamental questions must be asked before linking different types of data. For example: Is there a scientific basis for connecting the data sets? Are the data to be linked adequate and appropriate for addressing the issue? A useful framework for examining these questions has been presented by Thacker et al. (1996) in their description of a seven-step “hazard-exposure-outcome” axis that outlines the process of how an environmental hazard produces an adverse health outcome. In this process, Thacker et al. elucidate the steps whereby an environmental agent moves through the environment (hazard), enters a person (exposure), and produces an effect (health outcome). Associations made between hazard and health are not conclusive without considering actual exposure, although exposure is often estimated from hazard data.

Thacker et al. (1996) further describe the type of public health surveillance associated with each of these steps: *a*) hazard surveillance tracks the presence of an agent in the environment and environmental pathways (e.g., air, water) leading to the routes of exposure; *b*) exposure surveillance monitors exposure of the host to the agent (biomarkers of exposure), the distribution to a target tissue, and production of effects (biomarkers of effect); *c*) outcome surveillance follows clinically observable disease (Thacker et al. 1996). The linkage of two or more types of data provides a powerful tool but only if the steps in the process are taken into account.

## Data Issues and Implications of Data Inadequacies

Before examining statistical methods for linking various types of data, it is necessary to examine data sources that are available for tracking and linking hazards, exposure, and health effects. Fundamental factors that provide confidence in the results of data linkage are data quality, appropriate use of the data, and consideration of data limitations. The quality of hazard, exposure, and HOD are diverse, and the uses and limitations of data outside of its original purpose are not yet well defined.

### Hazard data.

Hazard data provide information about the presence and quantity of contaminants in environmental media. A hazard has the potential for harmful effects, but its presence alone may not be sufficient to produce an adverse effect in a population. Most environmental data collection by federal and state environmental agencies is mandated by legislation such as the Clean Air Act (1970), Clean Water Act (1977), and Safe Drinking Water Act (1974), and data are used for regulatory purposes. Examples of environmental data include the Toxic Release Inventory (TRI; http://www.epa.gov/tri/), criteria air pollutant data (ozone, sulfur dioxide, particulate matter), pesticide exposures, and the Safe Drinking Water Information System (SDWIS; http://www.epa.gov/enviro/html/sdwis/). Data analysis is often limited to comparison of each data point with an environmental standard or guideline, and regulatory action is triggered if the data point exceeds the standard. Standards are not available for all environmental contaminants, and those that do exist are extrapolated primarily from toxicologic studies. Standards are developed for regulatory purposes and include uncertainty and safety factors that are meant to be protective of public health; exceeding the standard does not predict health outcomes in the population.

Hazard data alone are not measures of individual exposure; in some cases, certain types of hazard data; for example, monitoring contaminants in drinking water, may contribute to the characterization of population exposure. Table 1 lists limitations in the use of hazard data for EPHTN.

### Exposure data.

Exposure data are the essential link between environmental hazards and health outcomes. Optimally, exposure data include biomarkers such as a compound or its metabolite(s) in a biologic sample (e.g., blood, urine, hair, fat). Levels of agents in blood or urine are not necessarily proportional to environmental concentrations. Additionally, factors such as exposure level, route of exposure, frequency and duration of exposure, baseline health status, behavioral factors, and genetics influence the internal dose of the compound in an individual.

Exposure data represent the largest data gap for EPHTN. Ideally, exposure data would be available at the individual level, but very few of these types exist. Childhood lead levels are one of the few nationwide exposure data sets. The CDC initiative to monitor exposure of the general population to 116 environmental chemicals through biomonitoring as a part of the National Health and Nutrition Examination Survey (NHANES) is providing essential baseline exposure data (CDC 2003b). However, there are no other systematic biomonitoring programs that provide nationwide exposure data. Data from sporadic, single-event biomonitoring that occurs in response to specific incidents or investigations are not widely available. Another limitation is the rapid metabolism and clearance of many compounds of concern. Because of the limited availability of exposure data, these parameters are often estimated rather than measured. Exposure modeling and assessments, as used in regulatory risk assessment, are meant to be protective of public health and therefore use conservative assumptions that overestimate actual exposure (e.g., consumption of 2 L/day of water for 70 years; U.S. Environmental Protection Agency 1989). Multiple routes of exposure and chemical mixtures are difficult to estimate. Individual factors that modify exposure, such as age, race, gender, time near the hazard, behavioral factors, and genetics, are often not incorporated into the exposure assessment models. Inexact exposure estimates limit the ability to ascribe health outcomes to a contaminant.

The lack of exposure data further complicates issues because the level of exposure influences the appropriate health end point. At high exposure levels, health effects are observed within a relatively short time frame and are often documented in the scientific literature. Low-dose chronic exposures present greater challenges: health outcomes may not be known; effects of low-dose exposures are not observable for years or decades; effects may require repeated exposures over time; multiple agents may need to interact simultaneously or sequentially; and the effect may occur only in sensitive subpopulations or those with existing health conditions. Table 1 lists additional limitations in using exposure data for environmental public health tracking.

### Health outcome data (HOD).

Adverse health events such as morbidity and mortality are the outcomes of interest in efforts to associate environmental hazards and exposure with effects in humans. Of particular interest are asthma, birth defects, cancer, and sequelae of lead and pesticide poisoning/exposure. Health events may be identified and evaluated at the individual level, as is the case in traditional epidemiologic studies, or may be aggregated for populations, such as those available from local, regional, and national surveys. The uses and limitations of population-based aggregate data can differ from those of individual-level data, and this affects the use of each type of data.

EPHTN efforts focus on the use of existing (secondary) data rather than on the generation of new data (primary). Sources of such data include local, regional, and national health surveys such as the NHANES and the Behavioral Risk Factor Surveillance System, and health events and reportable disease registries such as those for cancer—the National Cancer Institute’s (NCI) geographic information system (GIS) web site (NCI 2004b)—and birth defects. Other sources include state vital records, hospital discharge data, emergency room data, and health insurance statistics. Several potential limitations must be considered when using secondary HOD, including those related to evolving or changing diagnostic criteria, misclassification, generalizability, measurement error, and completeness. Secondary HOD are also limited because many diseases/conditions are not reportable, resulting in incomplete information for many outcomes of interest. Further, confidentiality may prohibit access to the data at the level of detail that might be needed for correlation with an exposure, for example, address of an individual. Table 1 lists additional limitations in using HOD for EPHTN.

### Other relevant data (covariates).

The association of a disease with a risk factor in epidemiologic research requires more than observing corresponding fluctuations between the two factors. For example, examining the effect of air pollution as measured by criteria pollutants on the incidence of asthma must consider related factors. Other factors may include residence, proximity to known asthma-causing sources, socioeconomic status, age, race, and adherence to treatment regimens that may be related to incidence and hazard/exposure. These characteristics related to the factors of interest must also be considered and controlled. In epidemiologic research, strict control is not feasible, in the sense that these characteristics are not assigned by the researcher. Instead, important factors are observed, measured, and controlled in the analysis. Information regarding many of the standard covariates is routinely collected and made available through the U.S. census and surveys, including information regarding race, age, and sex distributions, as well as a variety of variables describing socioeconomic status, employment, and housing statistics, among others. These data are aggregated by specified units (e.g., state, county, census tract) and have the same limitations identified above for HOD. The decennial census data have limitations with respect to how current or complete the data are for a given time period. Migration is problematic, especially in estimating populations for small areas. Nevertheless, these data provide important information regarding population characteristics necessary for establishing denominator estimates and associating hazard/exposure with health outcomes.

## Statistical Framework

Data limitations directly affect the outcome of statistical analyses and may at times limit the level of analyses that might be performed with a given data set. The lack of consistency in the quality of geocoding in the U.S. spatial infrastructure provides an example of such a limitation. The street reference files in urban areas may be of very good quality, whereas in rural areas they may have limited accuracy. Consider the impact of this varying quality on geocoding when comparing urban and rural areas. If unmatched cases are omitted, urban/rural differences may be attributed to geographic covariates rather than the inaccuracy of geocoding. Alternatively, if unmatched cases are allocated to the geocenter of the spatial area, distances from a putative exposure are still less accurate in the rural area than in the urban area. Current statistical methods do not account for this variability in quality of geocoding. These limitations highlight the need to closely examine available data sets and determine the appropriate use of the data as they exist and the assessment of data quality before more complex statistical analyses are applied. Additionally, data are collected from disparate sources (e.g., U.S. Environmental Protection Agency, CDC, U.S. Census Bureau, state and local health and environmental agencies) for a wide variety of purposes (regulatory vs. surveillance). These data sources have varying levels of quality related to completeness, consistency of definitions, accuracy, and timeliness. Further, the locations (i.e., state, county, ZIP code, census tract, point source) of data collection can be inconsistent or “misaligned.”

The complex relationship between exposure and health outcome requires careful consideration of the type of statistical model that should be used to represent this relationship. Ecologic bias is a potential problem when hazards ascertained from data aggregated over a larger geographic area are attributed to individuals within the area. It is fairly common to see analyses in which an aggregate measure of socioeconomic status for a ZIP code is assigned to each individual within the ZIP code and subsequently analyzed by methods ignoring the aggregation. The bias will decrease with smaller geographic areas and may be decreased by measuring confounding variables. But all the confounders may not be known, so whether bias has been eliminated is generally unknown (Wakefield and Elliott 1999). In general, data containing variables measured at individual and aggregate levels should be analyzed by means of hierarchical models that better describe the data and account for the limitations of these data in terms of the potential for ecologic bias.

Wakefield and Elliott (1999) and Banerjee et al. (2004) review a variety of statistical methods appropriate for the analysis of environmental and health data as well as the health/environment relationship. These methods attempt to realistically represent the hazard–exposure–disease process while also considering measurement issues. We organized these into three groups generally representing increasing complexity of study design.

The first group is composed of descriptive analyses and includes tracking trends (surveillance), temporally and spatially, of hazard, exposure, and HOD. These analyses provide information on recognizing and defining the scope of a problem, describing trends, generating baselines, and comparing changes in temporal and spatial indices of health and environmental data. GIS methods have been developed for exploratory data analysis and identification of space–time patterns.

The second group is ecologic analysis, which includes more advanced methods of spatial analysis and involves observational studies that provide information on the relationship between hazards and health outcomes. These hierarchical models can accommodate data from several levels such as cancer registry case point data and such regional data as poverty at the census, block group level. Ecologic analysis is appropriate for hypothesis generation and provides essential information needed before moving on to the more rigorous study designs in group three.

Group three consists of etiologic research using full-scale epidemiologic studies to test hypotheses describing the relationships between environmental exposures and a health outcome. These studies require high-quality, well-defined data and complex statistical methods that account for issues present in the data.

## Group 1:Tracking and Trend Analysis

### Time trends.

Examining time trends in HOD is helpful in identifying disease clusters. An example is the observation of birth defects (rare limb malformations) within a short time among women who took thalidomide for morning sickness. This cluster helped to identify thalidomide as a human teratogen (Taussig 1962). Trends identified through hazard surveillance are also important for characterizing background and changes in environmental contamination. Seasonal patterns are also identified through surveillance, for example, deaths from heat waves in the summer and pneumonia and influenza deaths in the winter season. Statistical approaches to the detection of clusters of disease include cell-count methods that compare observed with expected counts of events (Knox 1964; Openshaw et al. 1987, 1988), adjacency methods that examine whether areas of high rates of disease are likely to be adjacent to other high-rate areas (Moran 1948), and distance or nearest-neighbor methods that compare physical distances between cases to expected distance (Besag and Newell 1991; Cuzick and Edwards 1990; Mantel 1967). Alexander and Boyle (1996) provide extensions to the basic methods. Other statistical methods can be used to determine interarrival times between rare disease cases or to model seasonal patterns using time series methods, autoregression methods, and joinpoint regression (Kim et al. 2000). These trends are informative; however, confounding may prohibit correct interpretation. An example is an observation in prostate cancer incidence: A study was conducted to examine the prostate cancer incidence in a plant manufacturing triazine (MacLennan et al. 2002). Subsequent investigation of prostate cancer incidence indicated that the increase may have reflected increased prostate-specific antigen (PSA) screening rates rather than increased incidence of disease.

### Spatial analysis and geographic distribution.

Environmental and health data have very few commonalities; in many cases, the only commonalities of the data are the general geographic area and time frame. GIS is a useful tool for examining each type of data and one of the few methods that can be used to compare disparate health and environmental data. These include the “brush and link” exploratory data tools such as GeoDa (Anselin et al. 2004) and identification of space–time patterns as available in SaTScan software (SaTScan 2004) and the TerraSeer Space Time Intelligence System (TerraSeer 2004). The common denominator is the geocode for each type of data. Issues arise in reconciling the point of sampling and the location (and often lack of location) of individuals with the health outcome. Nonetheless, simple mapping of each data set and overlay of the maps can assist in targeting geographic areas. GIS software often comes with an array of tools to produce maps with impressive visual displays of data, overlay of disparate environmental and HOD, and conduct trend analysis. Because of this, caution is necessary to ensure that the maps are not misleading. This can occur when health and hazard data are mapped independently and there is confounding in the data that may lead the investigator to an inappropriate conclusion. For example, the potential confounding due to the latency period and ecologic fallacy must be considered in interpretation. Additionally, the residential address of a cancer case at diagnosis may not be a good proxy indicator for the lifetime environmental exposure given the mobility of the U.S. population. Geocoding methods need to be developed that allow better cumulative estimation of exposure that individuals might experience during their lifetimes. These should be available for regular or irregular points in residence or occupational histories.

Disease mapping summarizes the spatial and temporal–spatial variation in risk. When compared with spatial and temporal–spatial variation in exposure/hazard, one may get some disease etiology clues. The National Cancer Institute’s Cancer Mortality Atlas (NCI 2004a) and State Cancer Profiles (NCI 2004c) show county-specific maps that have been instrumental in identifying cancer disease patterns with the location of major industries by means of ecologic studies—nasal cancer in areas with furniture manufacturing (Brinton et al. 1977), lung cancer in counties with petrochemical manufacturing (Blot and Fraumeni 1976), bladder cancer where chemical industries were located (Hoover et al. 1975), and oral cancer in regions where snuff use was common (Blot and Fraumeni 1977). Comparing county-level health rates from maps requires that rates be adjusted for potentially confounding variables such as age, race, sex, and socioeconomic status. Elliott et al. (2000) discuss problems in the comparison of indirectly adjusted rates [standard mortality ratios (SMRs)] and directly adjusted rates [comparative mortality figures (CMFs)]. CMFs may be unstable if the stratum-specific rates are based on small numbers; however, they are unbiased estimates of the relative risk to the standard population. SMRs may be biased if proportionality assumptions are not met. Neither of these rates (SMRs, CMFs) adjusts for the overdispersion typically found in this type of data. Nevertheless, Breslow and Day (1987) observe that the use of SMRs or CMFs often leads to the same results. A clearer exposition of which summary measure to use would be a welcome addition to the literature. Maps of rates can be very deceptive if they are composed of different-sized units; for example, large units are overwhelming, and small units are often lost in interpretation. The microplot map (NCI 2004c) shows 95% confidence intervals for the units, and their intervals may overlap even though a choropleth map indicates a difference. Additionally, maps at different scales may give different patterns, further obscuring the interpretation. Observed rates are quite variable, especially when the expected rates are small, so maps of county-specific rates are smoothed by a variety of means to enhance the visualization of regional patterns of distribution. Smoothing methods need to consider and adjust for differences in the population size (denominator) for different geographic units. Methods to compute and visualize the spatial and temporal–spatial variability in disease/mortality controlling for such covariates as age, race, sex, and deprivation are currently available, as are extensions of the method to smooth the data and model heterogeneity and clustering of the area-specific effects in both the traditional approach and Bayesian framework (Banerjee et al. 2004; Wakefield and Elliott 1999).

**Figure 1:**
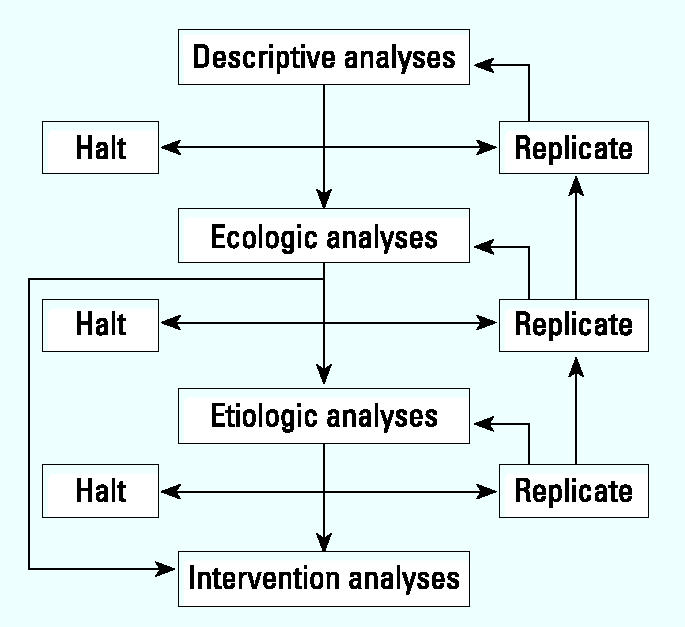
Decision tree for statistical framework.

These temporal, spatial, cluster, and exploratory methods have standard software to analyze the data. These should become standard procedures in health departments. Problems in interpretation will arise because of geographic resolution of disease and environmental data, data quality, confounding, and ecologic bias. Replication of the analyses in widely differing settings should provide scientists with background information to make the judgment as to whether to abandon the examination of a particular health outcome–hazard link, replicate the study, and/or proceed to an ecologic study if significant effects are apparent or sufficient evidence to warrant further investigation exists (see “Descriptive analysis” in Figure 1).

## Group 2: Ecologic Analysis

### Ecologic epidemiologic studies.

Ecologic studies describe the coexistence of risk factors with disease among and within populations. Aggregate exposure data are correlated with aggregate health data for each unit of observation, usually defined by geographic or administrative boundaries (e.g., city, county, state). Rates of exposure and rates of disease within each unit are known, but the exposure status of diseased individuals is not known. Analysis of ecologic studies can be conducted visually by interpreting the slope of a line plot of the exposure rate by the disease rate for each unit or using the correlation coefficient, *r*, as a measure of association. In ecologic trend analyses, changes in rates of exposure are correlated with changes in rates of disease over time. The most salient limitation of these studies is ecologic bias, resulting from spuriously ascribing aggregate-level observations to individuals. These studies are useful for generating hypotheses, for assessing the impact of community-level interventions, or for initial evaluation of suspected associations, although more rigorous studies are necessary to support a causal relationship between exposure and disease.

### Geographic correlation studies.

These studies model the interrelationships of hazard, exposure, and health over time and space. The objective is to relate environmental variables to disease and control for other factors such as life style (Banerjee et al. 2004; Wakefield and Elliott 1999). Poisson regression provides the framework for modeling the rates for rare diseases, whereas binomial regression or survival analysis is suitable when disease is more common. Counts or rates of events are described as a function of exposure, space, time, demographics, and other variables. The models are hierarchical when hazard data are used and are thus subject to ecologic bias. Spatial correlation in the data should be anticipated. For example, screening for PSA is likely to be correlated among counties when the screening initiative is directed toward a large area. Overdispersed data are an additional problem. The methods for estimating parameters relating hazard to health outcomes in these models are by means of likelihood methods and by Bayesian methods. Advantages of the likelihood methods include the readily available software and, in some cases, the incorporation of dispersion common in these data. Disadvantages include the difficulty of specifying complex covariance structures in the data, the unreliability of SMRs based on sparse data, and the problems of estimating variances of the relative risks. Bayesian methods applied to the data require the assumption of a prior distribution on these parameters from which a posterior distribution can be used to provide point (mean, median) and interval (95% confidence intervals) estimates for the parameters of interest. These estimates are obtained by means of Markov chain Monte Carlo methods using WinBUGS software (MRC Biostatistics Unit, Cambridge, UK; http://www.mrc-bsu.cam.ac.uk/bugs/welcome.shtml). Advantages of the Bayesian methods include the use of random effects to represent overdispersion, the availability of WinBUGS software for computation, and the provision of smoothed estimates of SMRs based on sparse data. Disadvantages of the Bayesian methods include the sensitivity of the estimates to the choice of the prior, and problems of convergence in complicated models (Elliott et al. 2000). Wakefield and Morris (1999) extend their analyses to accommodate spatial correlation and overdispersed data in the Bayesian analysis of the relationship between heart disease mortality and magnesium and calcium in water. Another example using Bayesian methods is a study measuring ozone levels at 10 fixed sites and emergency department visits—total visits and those for asthma by ZIP code—to assess the effect of ozone levels on pediatric emergency department asthma visits. In this example, the data for ozone and emergency department visits are “misaligned,” and before the data can be analyzed, a surface (kriging) for ozone from the site data must be developed so that an ozone level can be estimated for each ZIP code. Carlin demonstrates the use of hierarchical Bayesian models in solving this and other misalignment problems (Banerjee et al. 2004)

Multilevel models estimating hazard and health outcome effects and controlling for potential confounders and covariates provide hypothesis-generating information; however, they will likely require more refined hazard and disease data. Statistical methods are available but are not trivial to run and interpret, and the potential for ecologic bias remains. Replication of these studies in several areas should be guided by the descriptive analyses. Judgment of the scientific community is necessary to decide whether to halt studies at this level or, if significant effects and consensus of the scientific community indicate, then to design an in-depth study (etiologic research) (“Ecologic analyses” in Figure 1).

## Group 3: Etiologic Research Studies

Epidemiologic studies associate exposure in individuals to health outcome by means of case–control studies in rare diseases and by cohort studies in groups such as in occupational settings. Hertz-Picciotto (1998) summarizes a number of studies illustrating the difficulties of associating environmental exposure to the risk of disease, for example, the risk of serious illness (seizures and/or death) from drinking cider in Devonshire (Baker 1767); the risk of cholera associated with ingestion of water contaminated by fecal matter from infected patients (Snow 1855); poor mental development among children exposed to lead (Needleman et al. 1979, 1990; Needleman and Gatsonis 1990); and the many studies on the risk of health effects associated with air pollution (Hertz-Picciotto 1998). Statistical analysis of environmental data when appropriate exposure measures are of high quality is handled quite readily by multiple logistic regression in case–control studies and survival analysis methods such as Cox regression.

These stronger studies requiring better hazard/exposure and disease definition should benefit from results of the earlier studies. Statistical methods are available for hypothesis testing of the risk associated with exposure. The limitations of these studies are well known, but the problem of identifying weak effects in small populations remains. If the results of these analyses show promise and, in the opinion of scientists and policymakers, an intervention is both possible and desirable, then intervention studies in the form of community trials or policy decisions effecting current or new standards should be evaluated for impact. Intervention analyses may be implemented at early stages of study, after ecologic studies (“Etiologic analyses” in Figure 1).

## Discussion

The EPHTN is developing a framework to assess the impact of environmental agents on human health that will begin to fill in the “environmental health gap” described in the Pew Environmental Health Commission (2000) report. The environmental and health data that reside in federal and state health and environmental agencies hold a wealth of information if unlocked with appropriate linkages and analyses. The challenge is in the details for analyzing and linking disparate data in a scientifically sound manner while considering appropriate data uses and limitations. The magnitude of the endeavor dictates that we carefully articulate questions that might be reasonably answered with existing data in the near term and set an agenda to proceed to more complex and difficult questions.

The Thacker et al. (1996) model should be extended to account for exposures that effect only a susceptible population. Further, models specific for disease–exposure relationships explaining the fate and effect of an agent should be considered. Incorporation of policy changes and treatment interventions should also be considered. Guidelines on measurement and acquisition of data should be prepared. The Guide to Community Preventive Services (CDC 2004) program provides a model for how public health interventions and treatments could be represented in environmental situations.

The analytical framework presents groups of analyses to facilitate a progression from descriptive analyses to more complex linkages; this builds the foundation for etiologic research using epidemiologic studies.

The first group of analyses describes spatial and temporal distribution of hazards and outcomes independently and elucidates trends and relationships that can be further explored. These descriptive methods provide basic information to agencies and policymakers and suggestions for further studies. The second group of analyses focuses on ecologic studies that associate hazard with health outcomes using recently developed methods such as GIS spatial analysis, hierarchical models, and Bayesian methods. These methods address environmental and disease measurement issues, and experience with their use will generate hypotheses and a core set of analyses that may become the standard methods for linking health and environmental data. The third group relates exposure to outcome using traditional epidemiologic study designs to test hypotheses. These are research studies that should build on preceding descriptive and ecologic analyses.

The framework for analyses highlights the necessity for collaborations and partnerships. Data sharing is essential to EPHTN and requires overcoming the organizational and functional problems limiting collaboration between health and environmental agencies. Further, multidisciplinary teams with expertise in epidemiology, statistics, toxicology, environmental health, database management, GIS, and other areas will be required to ensure sound science and appropriate analysis of data. The EPHTN has established academic/agency partnership and designated Centers of Excellence to serve as a resource to state health agencies. As more complex linkages and analyses are conducted, greater statistical expertise will be needed in this initiative.

## Conclusions

The linkage of two or more types of data provides a powerful tool, but only if the steps in Thacker et al. (1996) “hazard-exposure-outcome” model are considered. The lack of exposure data is an impediment to more complex linkages. In the descriptive analyses, the lack of exposure data may be acceptable, but studies of more complex linkages will require more and better data. Additional efforts to generate exposure data, perhaps in partnership with public health laboratories, need to be formulated.

Statistical methods are available to link hazards and covariates to health outcomes; however, the appropriate uses and limitations of each data set must be taken into account. The analysis of hazard data and their linkage to health outcomes are subject to ecologic bias; use of smaller geographical area may minimize that bias. Newer methods such as GIS spatial analysis, hierarchical models, and Bayesian methods are promising but require experience and repeated use with various types of linkages before they become standard techniques. If used properly, statistical methods are available to begin analyses and linkage of environmental hazard, exposure, and health data that in turn will provide information to the public, policymakers, and the scientific community.

## Figures and Tables

**Table 1:** Uses and limitations of hazard data, exposure data, and HOD.

Uses	Limitations
Hazard data	
Regulatory compliance	Not representative of individual exposures
Standard setting	Gaps in geographic coverage of monitors
Policymaking	High percentage of nondetected values in data
Characterization of pollution sources	Sampling and measurement errors are often unknown
	Reflect current levels of pollutants
	Insufficient data quantity for trend analysis
	Objectives for monitoring vary across environmental media
Exposure data	
Indicator of individual exposure to a hazard	Data rarely available at the individual level
Required to link hazard with health outcome	Misclassification of exposure
	Difficult to account for multiple exposure pathways
	Exposure models based on assumptions and uncertainties not included in statistical analysis
	Lack of data amount, frequency, and duration of exposure
	Variability within populations impedes generalizing exposure
	Difficult to reconstruct past exposure
Health outcome data	
Describes health status of populations	Data completeness
Describes distribution and frequency of disease	Misclassification of disease
	Generalizability to population
	Confidentiality issues (HIPAA*^a^*)
All three types of data	
	Completeness of records
	Timeliness of reporting
	Availability of access to data
	Geographic resolution of the data (scale)
	Frequency of data collection
	Lack of data collection standards

a HIPAA, Health Insurance Portability and Accountability Act of 1996 (1996).
